# Infection by *Leptospira* spp. in Cattle in a Tropical Region, Rio de Janeiro, Brazil

**DOI:** 10.4269/ajtmh.14-0519

**Published:** 2015-01-07

**Authors:** Camila Hamond, Gabriel Martins, Walter Lilenbaum, Melissa Pinna, Marco Alberto Medeiros

**Affiliations:** Laboratory of Veterinary Bacteriology Department of Microbiology and Parasitology Universidade Federal Fluminense Niterói-RJ, Brazil E-mails: camilahammond@gmail.com, gmartins@id.uff.br, and mipwalt@vm.uff.br; Department of Preventive Veterinary Medicine and Animal Production Universidade Federal da Bahia Salvador-BA, Brazil E-mail: melissahp@ufba.br; Bio-Manguinhos Oswaldo Cruz Foundation Brazilian Ministry of Health Rio de Janeiro - RJ, Brazil E-mail: medeiros@bio.fiocruz.br

Dear Sir:

We read with interest the study reported by Ayral and others^1^ entitled “Distribution of *Leptospira* serogroups in cattle herds and dogs in France.” Ayral and others^1^ concluded that the inventory of infecting *Leptospira* serogroups revealed that current vaccines in France are not fully capable of preventing leptospirosis. In total, 394 cattle were diagnosed with clinical leptospirosis, and the results suggested infection by serogroups Australis (43%), Sejroe (33%), and Grippotyphosa (17%).

We share our experience with bovine leptospirosis in Brazil and compare it with the scenario described in France. Infected cattle are commonly asymptomatic and may shed the bacterium in urine for long periods.^2^ Serology by microscopic agglutination test (MAT) is recommended as the primary diagnostic tool, although it is considered reliable only at the serogroup level.^3^ Although useful for a diagnosis at the herd level, serology is not adequate for the individual detection of carriers,^4^ which may impair control programs.^5^ For individuals, a rapid, sensitive, and reliable diagnostic tool, such as polymerase chain reaction (PCR), is required.

In a study conducted by our group in 2013, 208 cows were randomly selected in a slaughterhouse located close to Rio de Janeiro, Brazil. After clinical pre-slaughter examination, the animals were considered free of signs of leptospirosis. Blood samples (208) for serology (MAT)^3^ and urine samples (199) from direct puncture of the bladder for PCR-*lipL32*^5^ and culture were obtained. Serology showed that 77 of 208 (37%) animals were reactive, with serogroup Sejroe by far the most frequent (62.3%) followed by Javanica (7.8%), Icterohaemorrhagiae (6.5%), Grippotyphosa (3.9%), and Tarassovi (3.9%). Urine PCR showed that 67 of 199 animals (33.6%) were positive. Additionally, 10 isolates were obtained in pure culture, including serogroups Sejroe, Shermani, and Grippotyphosa with 2 isolates each and Sarmin, Tarassovi, Autumnalis, and Panama with 1 isolate each. These results showed a high rate of asymptomatic shedding in cows. Members of serogroups Grippotyphosa, Autumnalis, and Panama are usually associated with environmental contamination and maintained by wildlife species. Serogroup Sejroe is the most common in ruminants worldwide as well as in Brazil.^6^ Serogroups Sarmin, Shermani, and Tarassovi have never previously been reported from cattle in Brazil and may have been neglected as agents of bovine leptospirosis.

Serogroup distribution differed between Brazil and France. In our study, both seronegative and seroreactive cows were shedding leptospires. Therefore, the direct detection of leptospires in urine by culturing or PCR is an important tool for the success of control programs in cattle. Additionally, an active surveillance approach should also include asymptomatic animals, because they may shed the bacterium to the environment and consequently, play an important role in the epidemiology of leptospirosis.
